# Tools of the trade: MicroCT reveals native structure and functional morphology of organs that drive caterpillar–ant interactions

**DOI:** 10.1038/s41598-020-67486-5

**Published:** 2020-06-29

**Authors:** Dipendra Nath Basu, Krushnamegh Kunte

**Affiliations:** 1 National Centre for Biological Sciences, Tata Institute of Fundamental Research, GKVK Campus, Bellary Road, Bangalore, 560065 India; 2 SASTRA University, Thanjavur, Tamil Nadu 613401 India

**Keywords:** Entomology, X-ray tomography

## Abstract

Caterpillars of many lycaenid butterflies are tended by ants that offer protection from predators and parasitoids. Specialized structures such as glands, ciliary organs and chitinous ornamentation in caterpillars play key roles in the underlying tactile, acoustic, and chemical communication between caterpillars and ants. Although the ecological, evolutionary, and behavioural aspects of these interactions are well studied, the mechanisms (i.e., the functional morphology) that drive the specialized interactive organs are poorly characterized. We used advanced X-ray microtomography (MicroCT) to delineate internal, native morphology of specialized larval dew patches, nectar glands, and tactile ciliary organs that mediate interactions between *Crematogaster* ants and caterpillars of the obligate myrmecophilous *Apharitis lilacinus* butterfly. Our non-destructive MicroCT analysis provided novel 3-D insights into the native structure and positions of these specialized organs in unmatched detail. This analysis also suggested a functional relationship between organ structures and surrounding muscles and nervation that operate the glands and tactile organs, including a ‘lasso bag’ control mechanism for dew patches and muscle control for other organs. This provided a holistic understanding of the organs that drive very close caterpillar–ant interactions. Our MicroCT analysis opens a door for similar structural and functional analysis of adaptive insect morphology.

## Introduction

Association with ants, termed myrmecophily, is widespread among plants^1–3^, mollusks^4^, and arthropods^5–8^. Butterflies in the family Lycaenidae show extensive ant associations^9–11^, with ~ 75% of the approx. 5,200 species in the family being myrmecophiles^12,13^. Myrmecophilic associations of lycaenid caterpillars have resulted in facultative or obligate relationships that range from mutualism to parasitism, mediated by a spectrum from mutual rewards to behavioural manipulation^14–18^. Along this spectrum of interactions, lycaenid caterpillars have evolved specialized morphological features such as a thick cuticular dermis that protects them from ant aggression^19^, extended thoracic legs^20^, dew patches and nectar glands (Newcomer’s gland) that provide sugary rewards to attending ants, and tactile organs that manipulate ant behaviour^21–24^. Additionally, pheromone-secreting glands, called pore cupola, mediate caterpillar–ant interactions^19^.

These specialized organs function sequentially to modulate ant behaviour. The pore cupola organs secrete appeasement pheromones to subdue aggressive behaviour of ants in close proximity^19,25^. The dew patches and nectar glands produce carbohydrate-rich secretions that are devised to reward the tending ants, although in some cases the secretions may be sucked in by the caterpillar before ants can take them up, i.e., they may only lure but not always reward the ants, which is part of the larval strategy of deceipt^19,22,26^. The tactile eversible organs are positioned near either anterior or posterior end of caterpillars^19,22,25^. Additionally, sensory tufts of the tactile organs assist in sensing trail pheromones or alarmones of ants, directing caterpillars to move towards ant aggregations^19,25^. The suite of these specialized caterpillar structures and behaviours modulate ant behaviour in favour of caterpillars, creating a protective, enemy-free space^27^. These interactions and protective umbrellas extend to pupal stages, where myrmecophilous interactions are largely mediated by acoustic and chemical means^28,29^.

**Figure 1:**
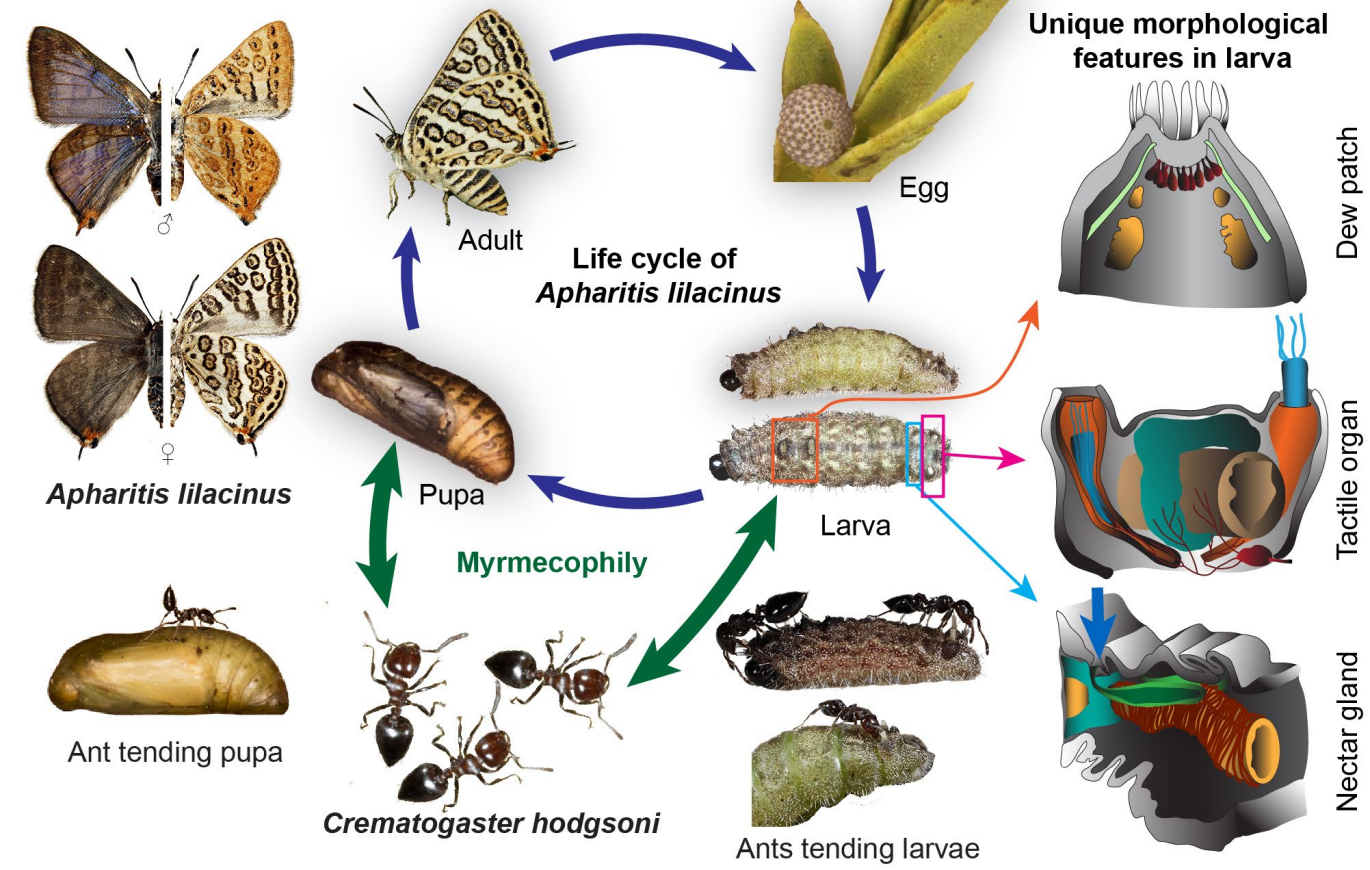
*Apharitis lilacinus* life cycle showing larval and pupal associations with *Crematogaster hodgsoni* ants. Structures of the specialized larval organs that mediate caterpillar–ant interactions are depicted on the right based on our MicroCT data (Figs. 2, 3, 4). Blue arrow in the nectar gland shows the external opening of the gland. The life stages, interactions, and MicroCT-derived 3D volumes of caterpillar and pupa of *A. lilacinus* are animated in Supplementary Movie 1. Image courtesy for egg: Nitin Ravikanthachari; pupa and ant-caterpillar/pupa interaction: Ashok Sengupta and G. S. Girish Kumar.

**Figure 2:**
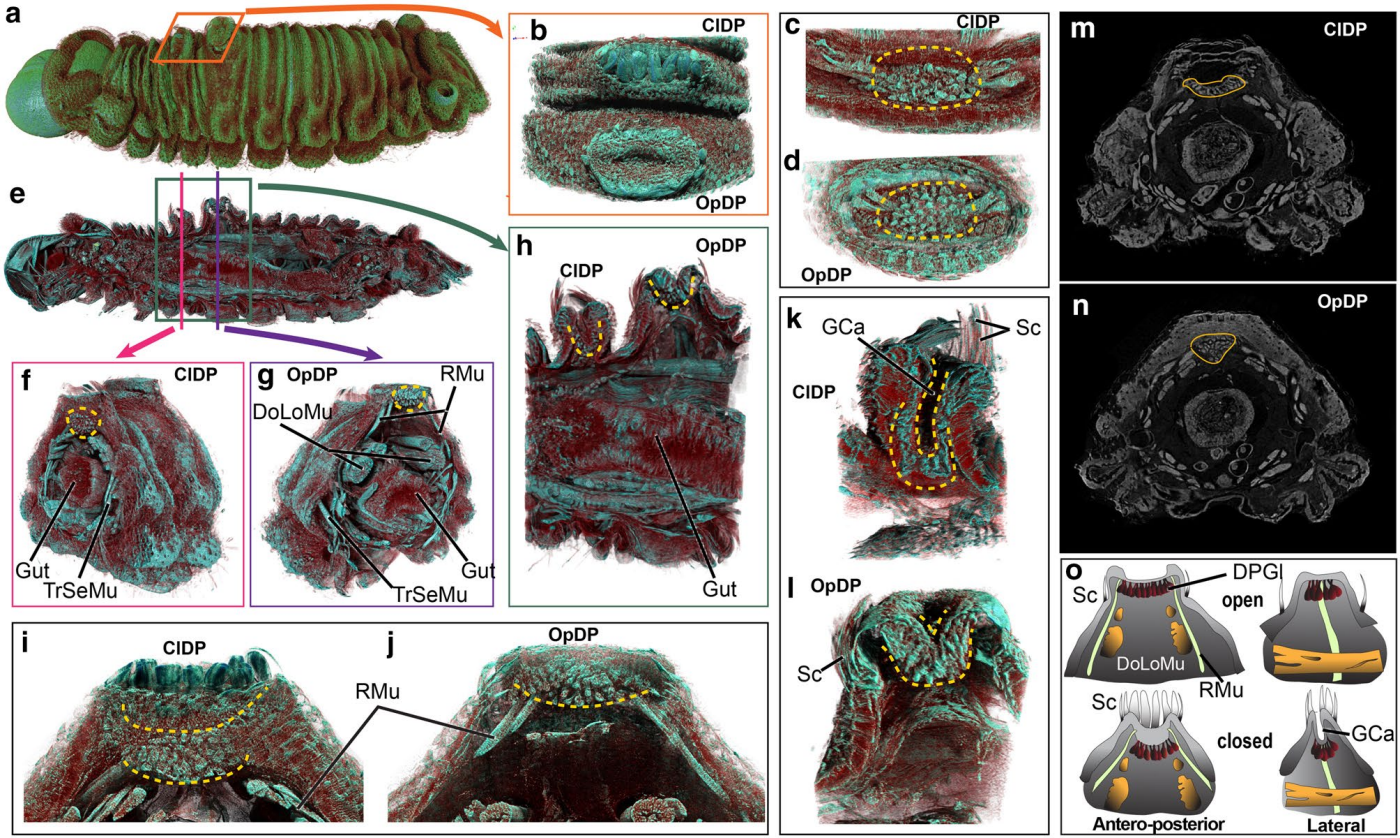
Dew patches of the *A. lilacinus* caterpillar. (**a**, **b**) external morphology in open (OpDP) and closed (ClDP) configurations of the dew patches; (**c**–**l**) depth-wise projections showing muscle organization and gut in relation to the dew patches (*Rmu* retractor muscles, *DoLoMu* dorsal longitudinal muscles, *TrSeMu* trans-segmental muscles, *Gca* gland cavity, *Sc* external scales); (**m**, **n**) original grayscale slice delineating gland positions (marked with yellow lines); (**o**) a diagram of the dew patches showing closed and open configurations of glands (*DPGl*: dew patch gland).

**Figure 3:**
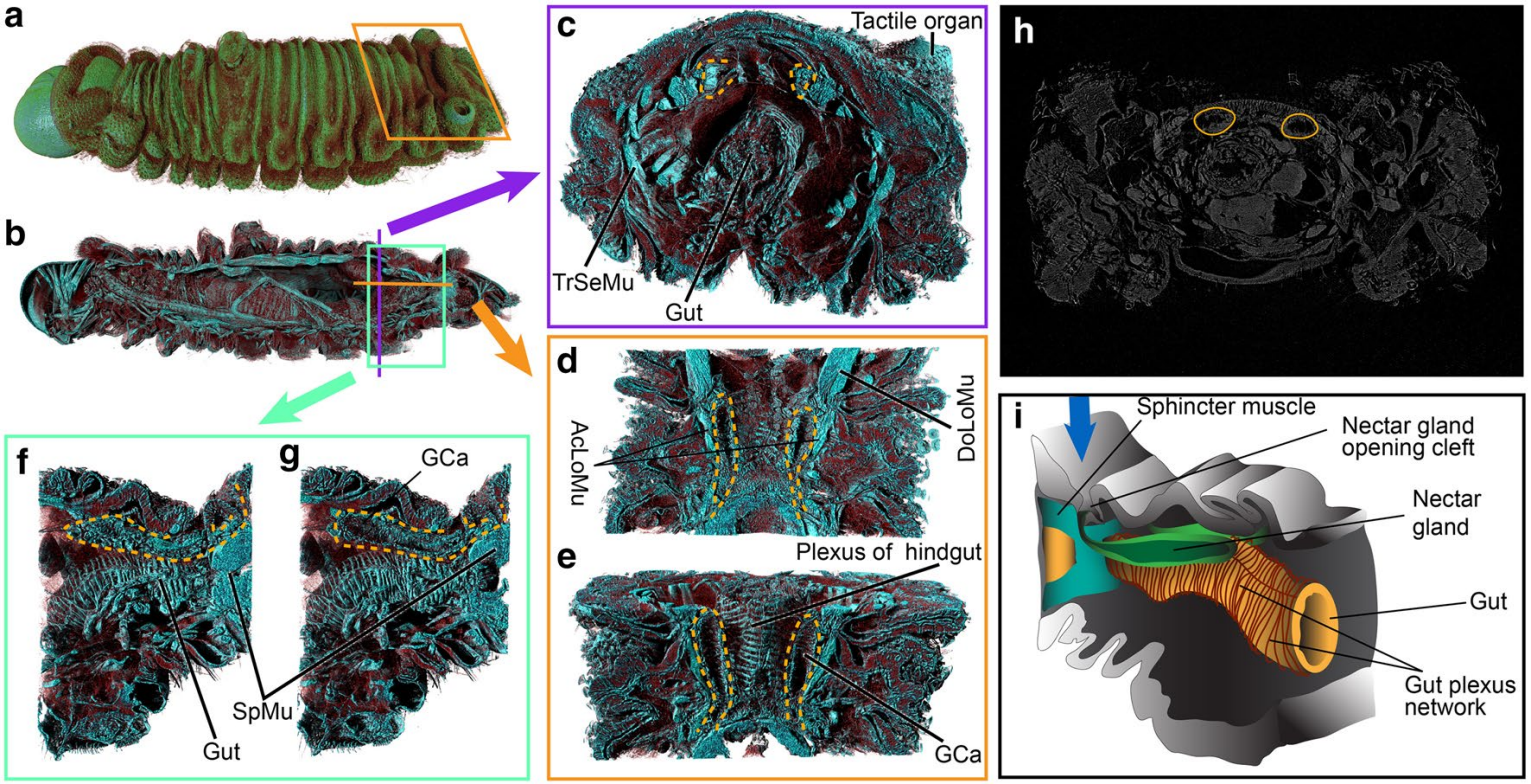
Nectar glands of the *A. lilacinus* caterpillar. (**a**) External morphology; (**b**–**g**) depth-wise projections showing internal organization of muscles (*SpMu* sphincter muscle, *AcLoMu* accessory longitudinal muscles, *DoLoMu* dorsal longitudinal muscle) and gut in relation to the nectar glands (marked with yellow dashed lines) and their temporary reservoir or gland cavity (Gca); (**h**) original grayscale slice delineating nectar glands; (**i**) a diagram of the nectar gland in relation to gut and sphincter muscle. Blue arrow points to the external opening of the gland.

**Figure 4:**
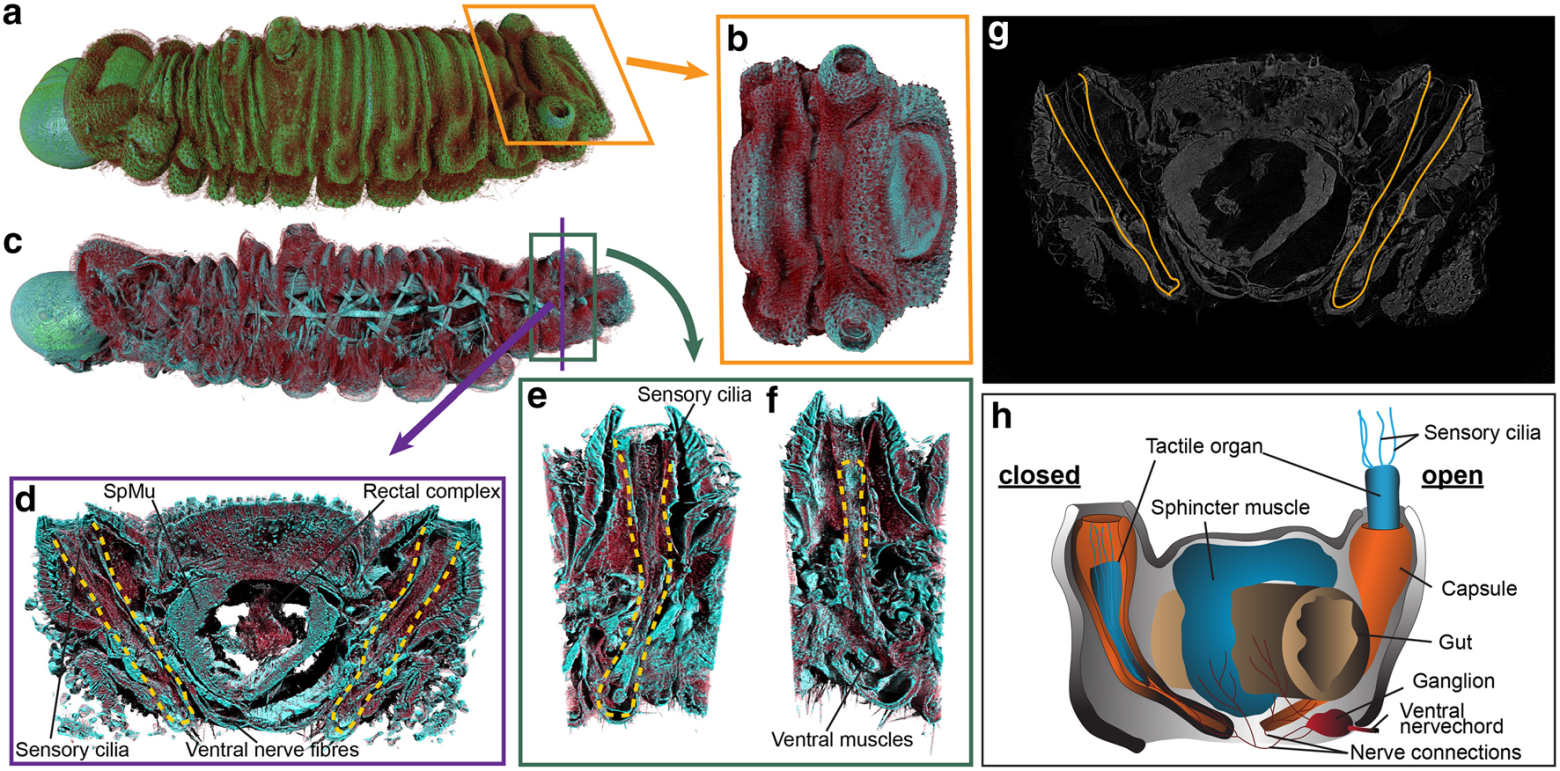
Tactile organs of the *A. lilacinus* caterpillar. (**a**, **b**) External morphology; (**c**–**f**) depth-wise projections showing internal organization of muscles (*SpMu* sphincter muscle), nerve fibres, and gut, in relation to the tactile organ chambers (marked with yellow dashed lines); (**g**) original grayscale slice delineating tactile organs; (**h**) a diagram showing open and closed configurations of tactile organs along with relative positions of gut, sphincter muscle, and the last abdominal ganglion.

The evolution of these organs across Lycaenidae^12,15^ with respect to their roles in ecological^30,31^ and behavioural interactions^18,27–29^ has been explored to a considerable extent. However, the detailed internal structures or functional morphology of these organs is poorly characterized, barring some preliminary studies with limited tools^19,22^. The interaction of these organs with surrounding anatomical systems is also unknown. To address these limitations, we used advancements in microtomography (MicroCT), which enable fine understanding of internal and functional morphology^32,33^. MicroCT provides high resolution and three-dimensional reconstruction that enables detailed quantitative and qualitative characterization of hard as well as soft tissue in their native states, without requiring dissection or histology^34–37^. Therefore, we used MicroCT to characterize the internal morphology of specialized organs in the early stages (caterpillar and pupa) of *Apharitis lilacinus*, which has an obligate association with *Crematogaster hodgsoni* ants (Figs. 1 and S1a–c)^38^. Female *A. lilacinus* deposit eggs at the nest entrance of *C. hodgsoni* ants—sometimes on sand and away from plants. Caterpillars and then the pupae are completely dependent on ants from hatching to eclosion, and constantly attended (Fig. 1)^38^. Caterpillars do not feed on plant tissue at all, but only on regurgitated food provided by ants. It is possible that they also eat ant broods when available, but this needs to be confirmed. In any case, all stages of *A. lilacinus* have obligate association with ants, and they possess all the ant-associated organs described in obligate lycaenid myrmecophiles. Our MicroCT-assisted study revealed not only the fine structures of these organs, but also their functional morphology in relation to surrounding musculature and nervation, substantially advancing the understanding of the mechanistic basis of these caterpillar–ant interactions (Figs. 2a–o, 3a–i, and 4a–h; Supplementary Movie 1).

## Results and Discussion

The high magnification MicroCT scans provided sharp delineation of the specialized ant-associated organs, resolving structural features such as their native size and shape, gland mass, ducts, external orifices, and cuticular boundaries (Figs. 2a–o, 3a–i, and 4a–h; Supplementary Movie 1). The abdomen contained dew patches in the two anterior segments, and tactile organs and opening of nectar gland at the posterior end (Fig. S2a–c). It also revealed associated anatomical features such as musculature, major ganglia and neuronal connections at low magnification (Fig. S3a–f).

### A ‘lasso bag’ control mechanism operates the ant-appeasing dew patches

The two dew patches, which release sugary secretions to appease tending ants, were positioned on the dorso-median line of the second and third abdominal segments (Figs. 3a–i and S3a–f). We show both closed and open configurations of the dew patches (ClDP and OpDP, respectively, in Fig. 2a–o). The gland openings were lined with specialized elongated scales (Sc), which completely covered the gland cleft when closed. The eccrine or apocrine gland lobules lined the inner cleft of the dew patches in both configurations (Fig. 2c–d). Lateral view of dew patches showed connecting ducts from each glandular lobule (marked in yellow dotted lines in Fig. 2a–o) opening into an eversible cleft (GCa), which likely acts as a temporary reservoir. In the closed configuration, the cleft became narrower and deeper, and the gland lobules lining the cleft sunk further inside the body, compared to the open configuration (Fig. 2i–j). The opening and closure of the dew patches would be controlled by associated dorso-ventral retractor muscles (RMu), connecting the lateral walls of the cleft with the ventral body-wall, encircling visceral organs.

These arrangements suggest a ‘lasso bag’ control mechanism for the dew patches, similar to pore cupola^19^. The retractor muscles should constrict to expand the gland cavity, pushing gland lobules closer to the surface and enhancing the surface area for secretion. While closing, the muscles should relax, sinking the gland lobules inside the body, closing the gland cavity, and decreasing the surface area to minimize exudate release (Fig. 2a–o).

### Accessory longitudinal muscles control release and retraction of secretions from nectar glands that bait ants

Nectar glands feature prominently in obligate myrmecophilic caterpillars^39^. In *A. lilacinus*, they started at the junction of abdominal segments 5 and 6, and opened externally in a common cleft between segments 7–8, connected antero-laterally with flanking accessory dorsal longitudinal muscles (Fig. 3a–i). From the lateral view, the glands were parallel to the dorsal dermis anteriorly, and gradually bent upward towards the cleft opening. The nectar gland lobes were not clearly visible, but the single long, slender reservoir and ducts were resolved (marked in yellow-dotted lines in Fig. 3a–i).

The accessory muscles should normally constrict in response to ant palpation, possibly pushing contents of the gland reservoir into the cleft. The trans-segmental, accessory longitudinal, and sphincter muscles associated with the common cleft should control its opening, releasing the nectar gland secretions. The last abdominal ganglion is connected with all these muscles, which presumably orchestrates their actions to release or suck back nectar gland secretions while baiting the ants.

### Muscles and haemolymph pressure likely operate tactile organs that engage ants

The tactile organs, as the name suggests, mediate interactions primarily through tactile responses^22^, although pyriform glandular cells at the base of sensilla contribute to chemical communication by producing pheromones^19^, thus together facilitating multi-modal communication. In *A. lilacinus*, we observed the paired tactile organs on the eighth abdominal segment (also see Fig. S2a–c) with a tuft of terminal sensilla on a whitish ciliary body everted when ants palpated. MicroCT showed that the tactile organs were multi-layered, chitinous tubes, covered with a thick dermal layer, their external orifices being annular (marked with yellow-dotted lines in Fig. 4d–f). They spanned nearly the height of the body, continuing till the ventro-lateral side of the hindgut, and were lateral to the sphincter muscles (Fig. 4d–g). The tufts of sensilla on the ciliary body were partially resolved (Fig. 4d).

Ant palpations presumably induce eversion of ciliary bodies, which is likely controlled by increased haemolymph pressure and ventral muscles that are connected with the last abdominal ganglion (Fig. 4h).

### Other ant-associated morphology

Our MicroCT analysis revealed two other morphological features that contribute to myrmecophily of *A. lilacinus* caterpillars. Similar to other lycaenids^19^, *A. lilacinus* caterpillars had a thick, convoluted dermis (Fig. S3a–f) to protect against ant aggression. The pro-thoracic plate and endplate were also thick and chitinous (Fig. S3a–f), which presumably act as protective shields for the sensitive head and abdominal ends. Second, the foregut volume was remarkably small (Fig. S3a–f), which was potentially related to dependence on trophallaxis in the caterpillar, which obviate the heavy digestive machinery found in caterpillars that have to digest tough vegetation.

Finally, the pupae of obligate myrmecophiles interact with ants via substratum-borne acoustic signals that are produced by stridulating chitinous micro-ridges at the segmental ends^28,29,40^. However, in the obligate myrmecophilic *A. lilacinus* pupae, we did not observe stridulatory ridges in the high-resolution scan. Stridulatory ridges are perhaps highly inconspicuous in *A. lilacinus* pupae, or their ant associations are mediated purely by pheromones and other non-structural means. However, MicroCT delineated in fine detail all the standard external and internal morphological features of the pupa that are also seen in metamorphosed adults (Fig. S4a–f; Supplementary Movie 1).

The understanding of mechanisms underlying insect symbioses remains limited because their chemical and structural bases remain poorly characterized, often due to unavailability of adequate methods. Recently developed MicroCT techniques permit very fine characterization of internal structures of small organisms in native (natural) states, elucidating the physical bases of these adaptive behaviours and interactions in unprecedented detail. Our MicroCT data delineated both the external surface topology and fine internal anatomical organization of the caterpillar and pupa of the obligate myrmecophilous *A. lilacinus* butterfly. Additionally, this analysis suggested how the supporting musculature and nervation may operate these organs during interaction with host ants that protect and feed the caterpillars. This is the first time that the ant-associated larval organs that facilitate these interactions have been functionally characterized at micron levels.

Functional morphology of the specialized organs, interpreted from our MicroCT data, is instrumental in the ‘push and pull’ mechanism of ant interactions with *A. lilacinus* caterpillars. The functional synergy of these organs builds a stable myrmecophilous strategy in this butterfly species. Likewise, a comparative account of functional morphology of these organs in different myrmecophilous caterpillars from different points on the interaction spectrum—from opportunistic mutualism all the way to obligate parasitism—could significantly enrich our mechanistic understanding of these inter-order ant-lycaenid butterfly interactions. Such a detailed mechanistic understanding will provide valuable insights into the spectacular diversification of this super-diverse butterfly family.

Finally, this study opens up a door for other mechanistic studies of the functional morphology of hard and soft tissue that are critical in behavioural, ecological and developmental aspects of insect adaptations. Functional morphologies of insect adaptations have always been considered important but they have been difficult to study because of technological limitations. Applications of similar MicroCT analysis may include a wide spectrum from mechanics of soft tissue in insect larvae and relative organ development through insect metamorphosis, to predator–prey interactions and arms-races.

## Methods

### Specimen collection

*Apharitis lilacinus* is an obligate myrmecophile, whose caterpillars live initially in the ant nests and later under the bark of *Acacia* plants^38^. It is legally protected in India under Schedule II of the WildLife (Protection) Act, 1972. As per the research and collection permit from the Karnataka Forest Department (see Acknowledgements), we collected two caterpillars from Bangalore, and raised them at room temperature and humidity in a plastic box along with a secondary *Crematogaster hodgsoni* ant colony under an *Acacia* bark.

### Sample preparation

We standardized a previous sample preparation protocol for MicroCT scanning^34^, for our butterfly samples. As per our protocol, we fixed larval and pupal samples in a solution of Bouin’s fluid (saturated solution of picric acid in 95% 80 ml ethanol, 37–40% 15 ml formaldehyde, and 5 ml glacial acetic acid) for 24 h at 26 °C. After that, we kept the samples in water to rinse excess Bouin’s solution, and then gradually dehydrated them with serially diluted ethanol solutions (50–100% w/v) for 15 min in each stage. We then stained the samples from 100% ethanol solution with ethanol-dissolved 5% iodine for 24 h. We submerged the samples in ethanol to leech excess iodine in two stages, each lasting 30 min. We performed critical point drying (CPD) of the samples. We then mounted the samples using two methods that are appropriate for two types of MicroCT scanning: (a) low magnification scanning to study overall structures, for which we mounted samples surrounded by polyurethane foams on a standard double-sided tape as the base, on a brass holder, and (b) for high magnification scanning to study detailed internal morphology, we mounted specimens with Fevicol glue—a synthetic, thermoplastic resin made from Polyvinyl acetate (Pidilite Industries Ltd.)—on a wooden holder.

### X-ray microtomography (MicroCT scanning)

We performed tomographic scans at the NCBS Electron Microscopy Facility. We scanned the samples in a Skyscan1272 high-resolution microtomographer (Bruker MicroCT, Kontich, Belgium) with a Hamamatsu L11871_20 X-ray source and a XIMEA xiRAY16 16-megapixel camera with a 7.4 µm pixel size. We used the following scanning parameters for larval scans: (a) for low magnification scan: isotropic voxel size of 2.2 µm/pixel, source voltage 40 kV, the source current 250 µA, and image rotation scan 180° with a 0.55° rotation step, and scanned in three steps, which were combined into a single volume with a scan duration of 16 m: 46 s, and (b) for high magnification scan: isotropic voxel size of 0.65 µm/pixel, source voltage 40 kV, the source current 100 µA, and image rotation scan 180° with a 0.4° rotation step, and scanned in six steps, which were combined into a single volume with a scan duration of 1 h:0 m:16 s. We scanned the pupa with the following scanning parameters: (a) for low magnification scan: isotropic voxel size of 7.5 µm/pixel, source voltage 40 kV, the source current 250 µA, and image rotation scan 180° with a 0.6° rotation step, and scanned in two steps, which were combined into a 3D volume with scan duration of 8 m: 53 s, and (b) for high magnification scan: isotropic voxel size of 1.25 µm/pixel, source voltage 54 kV, the source current 85 µA, and image rotation scan 360° with a 0.4° rotation step, and scanned in five steps, which were combined into a 3D volume with a scan duration of 1 h:19 m:57 s. We used three Bruker MicroCT Skyscan system software (NRecon, Dataviewer, CTAn) to stitch scanned steps into single 3D volume, set threshold, reorient, shrink wrap region of interest (ROIs), and obtain the final datasets of cross-sectional images (2D slices).

### 3D rendering

We rendered the 3D volumes using CTVox (Bruker) (Figs. 2a–o, 3a–i, 4a–h, and S1a–c, S2a–c, S3a–f, S4a–f; Supplementary Movie 1). We rendered the pupal morphology using Dragonfly (ORS Inc.) to get enhanced surface contours. We generated sets of discrete transfer function ranges for pseudo coloration to best resolve external and internal structures based on innate signal and contrast of the grayscale slices.

### Illustrations

We prepared illustrations of ant-associated larval organs (Figs. 1, 2a–o, 3a–i, and 4a–h) by tracing on the original projections from volume data in Adobe Illustrator CS5 (Adobe Systems Incorporated).

## Supplementary information

Supplementary Movie 1

Supplementary file
