# Supplementary material for: Tools of the trade: MicroCT reveals native structure and functional morphology of organs that drive caterpillar–ant interactions
Source: Sci Rep. 2020 Jun 29;10:10593. doi: 10.1038/s41598-020-67486-5 (PMC7324400; doi:10.1038/s41598-020-67486-5)
Supplement: Supplementary file 2 — Supplementary file [file 41598_2020_67486_MOESM2_ESM.docx]

**Supplementary Movie 1.** 3-D rendering of the MicroCT data showing functional morphology and anatomy of early stages (caterpillars and pupae) of the *Apharitis lilacinus* butterfly, including the caterpillars’ specialized organs that drive their interactions with ants.

**Figure S1.** MicroCT-derived external morphology of the ant *Crematogaster hodgsoni* (pseudo-coloured). **a**: dorsal view; **b**: lateral view; **c**: anterior view of head. Scales represent 1mm.

**
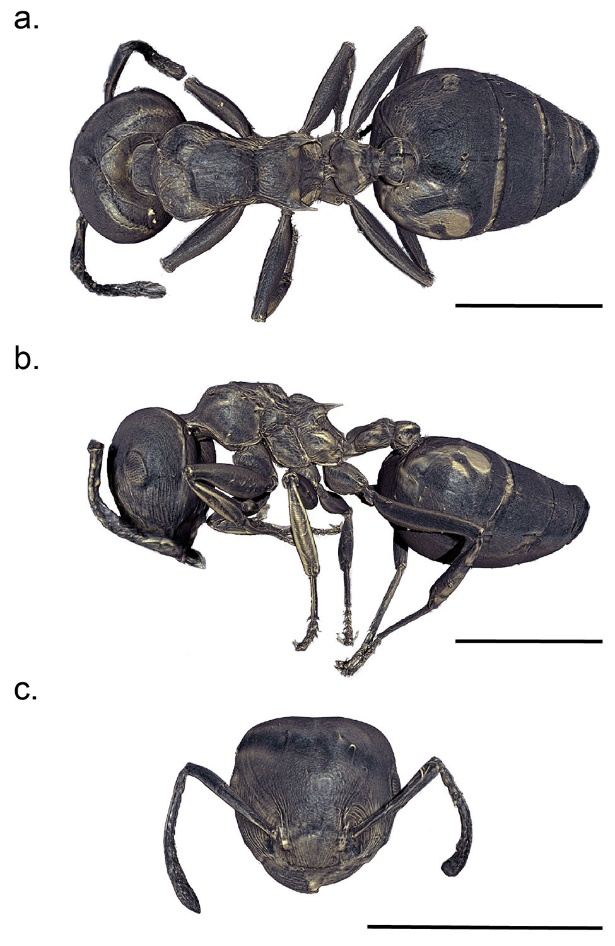
**

**Figure S2. Surface projections of the larval MicroCT showing external morphology.** MicroCT-derived 3D volumes of caterpillar surface are animated in Supplementary Movie 1. An: stub-like antenna; St: Stemmata; Max: Maxilla; PTC: prothoracic collar; ThLe: thoracic legs; DP: dew patch openings on the second and third abdominal segments; AbPrLe: abdominal prolegs; TO: tactile organ openings; Se: setae; Esu: epicranial suture; PTP: prothoracic plate; NGOp: nectar gland openings; EP: abdominal endplate; La: labrum; Fr: frons; Cl: clypeus; Cr: crochets on the mid abdominal prolegs; AnPrLe: anal prolegs.


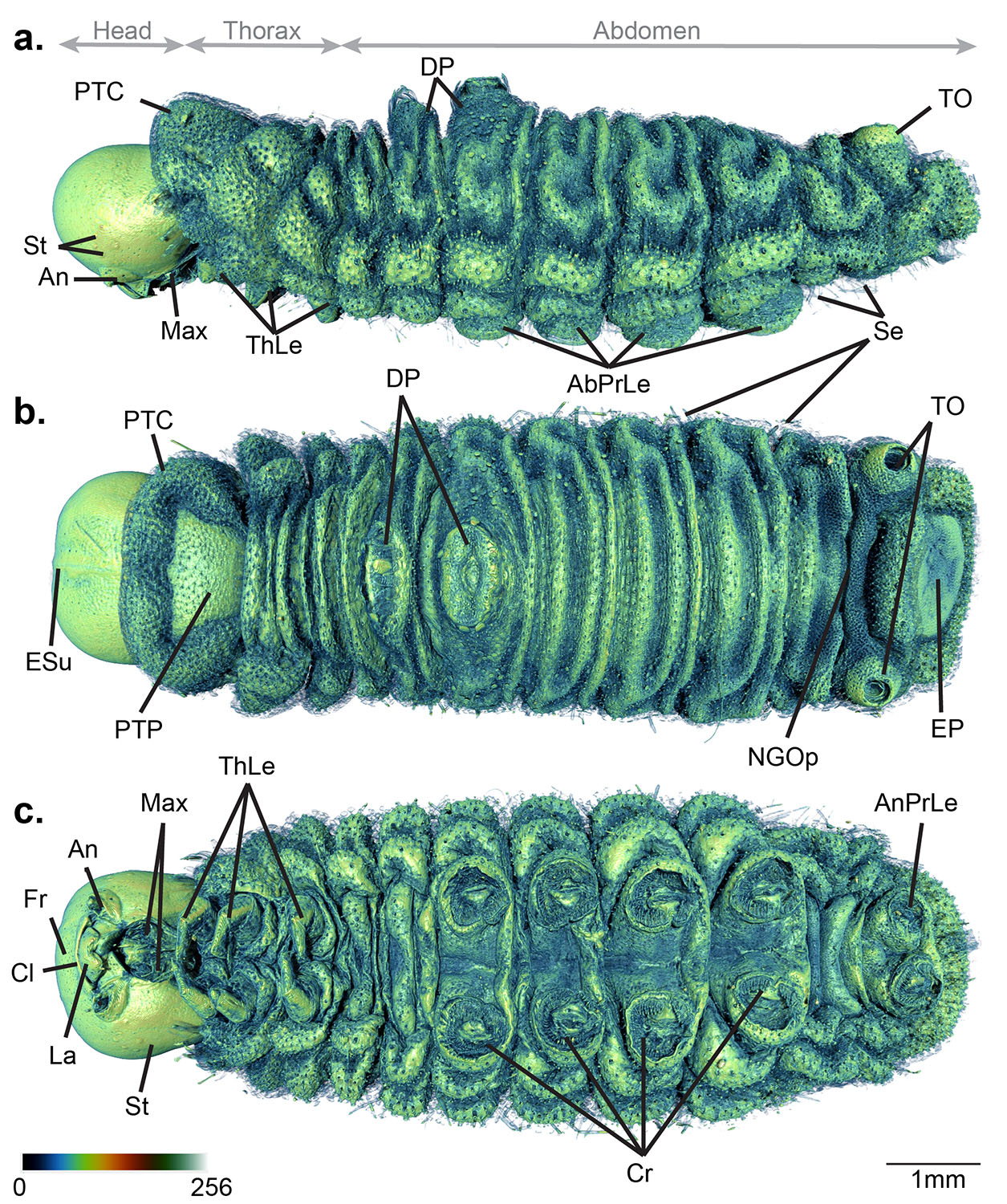


**Figure S3. Internal morphology of the caterpillar**. MicroCT-derived 3D volumes of visceral organs of caterpillar are animated in Supplementary Movie 1. Hmu: head muscles; VsMu: Visceral musculature; SpMu: sphincter muscle; NMu: neck muscle; ThCoMu: thoracic collar muscle; G(FG), G(MG) and G(HG): foregut, midgut and hindgut; SG: salivary glands; ProLeMu: proleg muscles; VeLoMu: ventral longitudinal muscles; DoLoMu: dorsal longitudinal muscles; CBMu: ciliary body muscles; ThLeMu: thoracic leg muscles; Rmu: retractor muscles; Go: gonads; NeG: nectar gland (Newcomer’s gland); EpMu: endplate muscles; CG: cerebral ganglia; ThG: thoracic ganglia; MdSe: mid septum; DP: dew patches; NeGOp: nectar gland opening; AbG: abdominal ganglia; AnOp: anal opening; TrSeMu: trans-segmental muscles.

**
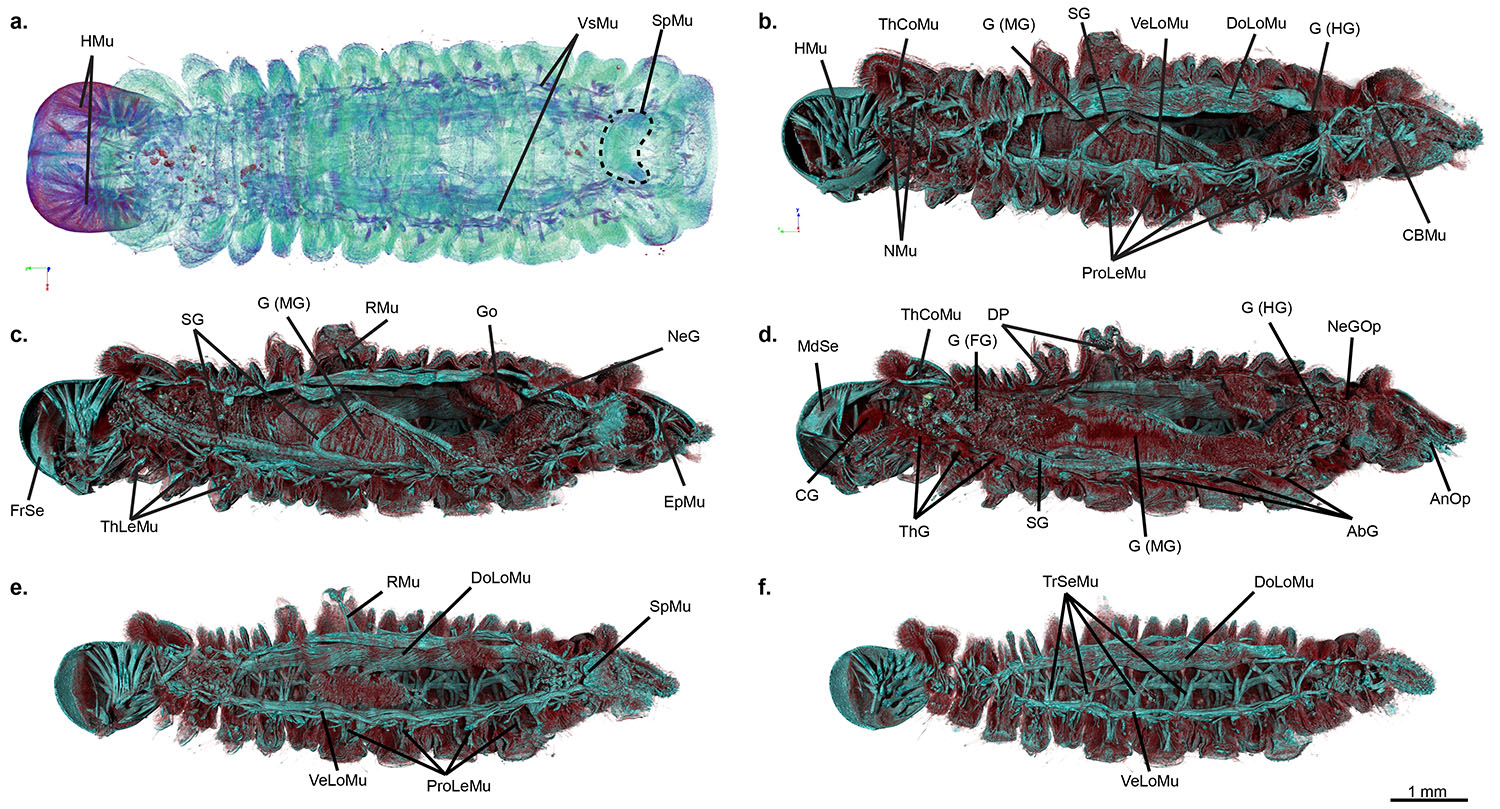
**

**Figure S4. External and internal morphology of pupa.** Also see Supplementary Movie 1. Hd: head; Th: thorax; WgF: wing fold; AbdSg: abdominal segments; Sp: spiracles; MeThSu: mesothoracic suture; PrGr: proboscis groove; AnGr: antennal groove; LgGr: leg grooves; WgFSu: wing fold suture; OpLo: optic lobe; DLM: dorsal longitudinal muscles; AbdViM: abdominal visceral mass comprising gut and meconium; An: antenna; Wg: wing case; CeGn: cerebral ganglion; ThGn: mesothoracic ganglion; ThMu: thoracic muscles; DoCiVe: dorsal circulatory vessel (heart); MpTu: Malpighian tubule; Aed: aedeagus (showing this was a male pupa).

**
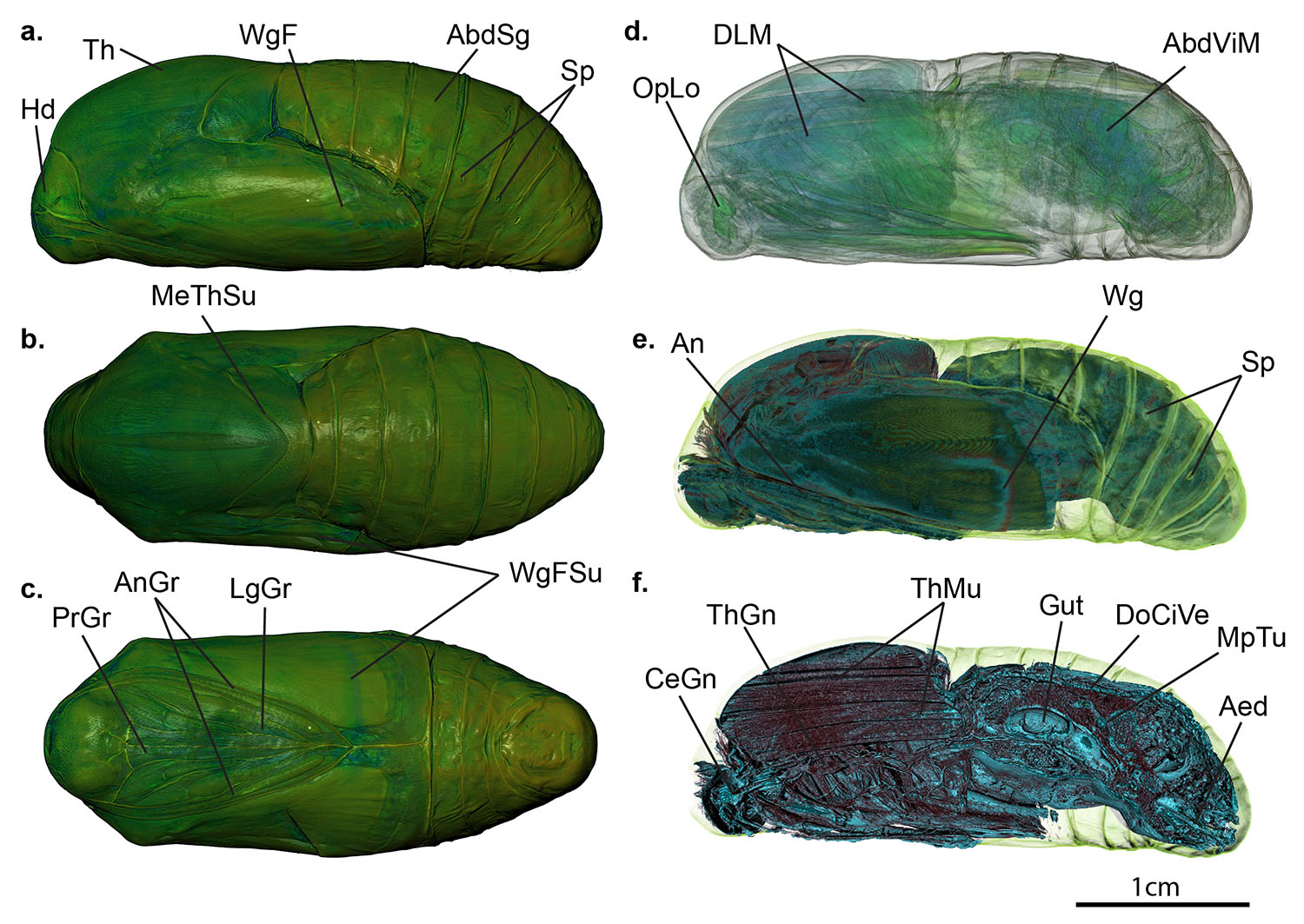
**
